# UV-B Radiation Impacts Shoot Tissue Pigment Composition in *Allium fistulosum* L. Cultigens

**DOI:** 10.1155/2013/513867

**Published:** 2013-03-31

**Authors:** Kristin R. Abney, Dean A. Kopsell, Carl E. Sams, Svetlana Zivanovic, David E. Kopsell

**Affiliations:** ^1^Plant Sciences Department, The University of Tennessee, 2431 Joe Johnson Drive, Knoxville, TN 37996, USA; ^2^Department of Food Science and Technology, The University of Tennessee, 2605 River Drive, Knoxville, TN 37996, USA; ^3^Department of Agriculture, Illinois State University, Normal, IL 61790, USA

## Abstract

Plants from the *Allium* genus are valued worldwide for culinary flavor and medicinal attributes. In this study, 16 cultigens of bunching onion (*Allium fistulosum* L.) were grown in a glasshouse under filtered UV radiation (control) or supplemental UV-B radiation [7.0 **μ**mol*·*m^−2^ *·*s^−2^ (2.68 W*·*m^−2^)] to determine impacts on growth, physiological parameters, and nutritional quality. Supplemental UV-B radiation influenced shoot tissue carotenoid concentrations in some, but not all, of the bunching onions. Xanthophyll carotenoid pigments lutein and **β**-carotene and chlorophylls *a* and *b* in shoot tissues differed between UV-B radiation treatments and among cultigens. Cultigen “Pesoenyj” responded to supplemental UV-B radiation with increases in the ratio of zeaxanthin + antheraxanthin to zeaxanthin + antheraxanthin + violaxanthin, which may indicate a flux in the xanthophyll carotenoids towards deepoxydation, commonly found under high irradiance stress. Increases in carotenoid concentrations would be expected to increase crop nutritional values.

## 1. Introduction

Fruits and vegetables have varying levels of phytonutrients, in addition to vitamins and minerals. Two important classes of phytonutrients are carotenoid and chlorophyll pigments. The primary carotenoids found in leaf tissue of most plant species include zeaxanthin, antheraxanthin, violaxanthin, lutein, *β*-carotene, and neoxanthin [1]. Chlorophylls are the dominant pigments in plants and serve primary roles in photosynthesis. These compounds are very effective antioxidants and help prevent certain types of cancers and aging eye diseases like macular eye degeneration [2, 3]. However, the chemical structures of these pigments also give them the ability to donate electrons and effectively become prooxidants under certain conditions [4]. *Allium* species contain chlorophyll and carotenoid pigments in shoot tissues [5] and also contain different levels of sulfur-containing compounds which prevent certain cancers [6]. While all higher plants contain chlorophylls and carotenoids, genetic variations for pigment accumulations exist both within and among plant species. Within any given crop species, there can be multiple landraces, accessions and cultivars, or, collectively, cultigens. These variations are important to advancements in plant development programs for increased nutrition, disease prevention, or other factors. However, cultigens will react differently under almost any given stress.

The absorption of light by chlorophyll and antenna pigments and the transfer of excitation energy to the reaction centers of PSII and PSI are the initial steps in photosynthesis. The photosynthetic apparatus has the ability to react to many different environmental stimuli, especially changes in light intensity. Under conditions of high light stress, photosynthetic systems are saturated, and excess energy needs to be diverted to avoid potential damage [7]. Carotenoids are unsaturated long chain polycarbons which protect the photosynthetic apparatus from high light excitation by quenching free radicals, functioning in nonphotochemical quenching, and dissipating excess thermal energy [7, 8]. The xanthophyll cycle, or violaxanthin cycle, is the mechanism by which plants regulate light energy available for photosynthesis. In intense light situations, violaxanthin is rapidly and reversibly converted to zeaxanthin, via antheraxanthin. Zeaxanthin is a direct quencher of chlorophyll excited states and can prevent photooxidative stress and lipid peroxidation [7].

Higher amounts of UV-A (380–320 nm) and UV-B radiation (280–320 nm) may influence the accumulation of plant compounds used to combat light stress. Carotenoid metabolites not only protect plants from excess UV radiation but also can protect humans from UV radiation when translocated to subdermal skin tissues [9]. What remains uncertain is the impact of increased UV radiation on growth and development and nutritional values of cultivated crops [10]. Previous studies have demonstrated impacts of UV radiation on plant performance, cellular structures, and pigment accumulations. In a study by Yuan et al. [11], 20 cultivars of wheat (*Triticum aestivum* L.) were grown under UV-B radiation stress to determine possible detrimental influences. Most wheat cultivars responded negatively to UV-B radiation; however, several cultivars showed increases in plant height and biomass. Structural changes like ruptured chloroplast envelopes have been noted in UV-sensitive rice cultivars (*Oryza sativa *L.) when exposed to UV stress [12]. Increases in UV radiation can delay flowering and harvest times among different cultigens of bush beans (*Phaseolus vulgaris* L.) [13]. Bush beans grown under UV radiation showed decreases in fruit size and yield when compared to cultivars not grown under UV radiation stress. Tomatoes (*Solanum lycopersicum* cv. DRW 5981) grown using UV-B blocking filters showed increases in lycopene and *β*-carotene, while fruits of the same variety showed decreases in lycopene, phytoene, and phytofluene when grown without UV-B blocking filters [14]. The tomato cultivar “HP1” accumulated more than twice the amount of lycopene in fruit tissues when grown under no UV-B radiation. Results from such studies demonstrate impacts on nutritional quality from excess UV radiation.

*Allium *species are valued worldwide for culinary flavor and medicinal attributes. Plants in this genus have been important to multiple cultures for centuries. *Alliums* have high levels of nutritionally important secondary plant metabolites which convey numerous health benefits. For example, bulb onions (*Allium cepa* L.) contain high levels of flavonoids [15], S-alk(en)yl-L-cysteine sulfoxides [16], and a variety of volatile antioxidant compounds [17]. However, no studies to date have measured the impact of UV radiation on the production of nutritionally important pigments in *Alliums*. *Allium fistulosum* is consumed, in part, for its shoot tissues as well as pseudostems. Carotenoid and chlorophyll compounds are present in the shoot tissues of *A*. *fistulosum* [5, 18]. Therefore, the objectives of this project were to examine both environmental and genetic responses to elevated UV-B radiation among a large subset of *A*. *fistulosum* cultigens. Responses were noted for plant height, shoot tissue biomass, photochemical efficiency (*F*_*v*_/*F*_*m*_), and concentrations of carotenoid and chlorophyll pigments in the shoot and pseudostem tissues.

## 2. Methods and Materials

### 2.1. Plant Culture

On December 16, 2008, seeds of 16 different *A. fistulosum* cultigens were sown in 15 cm pots holding soilless media in a glasshouse in Knoxville, TN USA (35°96′ N Lat.), which blocked UV-wavelengths (280–380 nm). The photosynthetically active radiation (PAR) in the glasshouse averaged 540 *μ*mol·m^−2^·s^−2^ (Apogee Nanologger model ANL, Apogee Instruments, Inc., Roseville, CA USA). The cultigens included eight accessions [PI 274254-05GI, PI 462345-05GI (Jionji Negi), PI 546343-90U01 (GA-C 76), PI 546228-06GI (Improved Beltsville Bunching), PI 280562-04GI (Pesoenyj), PI 436539-06GI (Zhang Qui Da Cong), PI 462357-06GI (Shounan), and G 30393-06GI] from the USDA-ARS National Plant Germplasm Repository (Geneva, NY USA); four cultivars (“Long White Bunching,” “Feast, Performer,” and “Parade”) from Seedway, LLC (Hall, NY, USA); and four cultivars (“White Spear,” “Evergreen Hardy White,” “Deep Purple,” and “Ishikura Improved F1”) from Johnny's Selected Seeds (Winslow, ME, USA). The seedlings were watered daily for the duration of the experiment. On January 10, 2009, the seedlings were thinned to two plants per pot and fertilized with a nutrient solution containing (mg·L^−1^): N (105), P (91.5), K (117.3), Ca (80.2), Mg (24.6), S (32.0), Fe (0.5), B (0.25), Mo (0.005), Cu (0.01), Mn (0.25), and Zn (0.025) [19]. Each pot was fertilized once a week for the duration of the experiment with 100 mL of nutrient solution. The experimental design was a split plot arranged as a randomized complete block. UV-B treatments were the main plots, and *A. fistulosum* cultigens were the subplots. Six individual plants per cultigen composed a replication, with four replications randomly assigned to each UV-B treatment.

Supplemental UV-B radiation (313 nm) was provided by banks of commercially available UV-B 313 lamps (Q-Panel Lab Products, Cleveland, OH, USA), and treatment began on January 27, 2009 delivering 7.0 *μ*mol·m^−2^·s^−2^ (2.68 W·m^−2^) of UV-B (Spectroradiometer Model SPEC-UV/PAR, Apogee Instruments, Inc., Roseville, CA, USA) to the treated plants. To control pests in the greenhouse, the beneficial insect species of *Hypoaspis miles *and* Neoseiulus cucumeris* were used to control thrips, while *Orius insidiosus* was used to help control aphids. These insects were first released on January 23, 2009, and were released every two weeks thereafter.

On March 3, 2009, all of the bunching onion cultigens were harvested. Six plants were harvested from each replication. Fresh weights and plant heights were taken and averaged for each replication. One measure of *F*_*v*_/*F*_*m*_ was taken from each of the harvest plants at the midpoint of plant height using a modulated fluorometer (OS1-F1 Modulated Fluorometer, Opti-Sciences, Hudson, NH, USA). The *F*_*v*_/*F*_*m*_ value is an indication of photoinhibition and overall plant health. All plants were harvested, and pseudostem and leaf tissue were separated. The samples were immediately placed in a −20°C freezer before being moved to a −80°C freezer within 8 h.

### 2.2. Pigment Extraction and Determination

Tissue pigments were extracted according to Kopsell et al. [20] and analyzed according to Kopsell et al. [5]. The samples were freeze-dried and ground with a mortar and pestle with liquid nitrogen. A 0.10 g subsample was rehydrated with 0.8 mL of ultrapure H_2_O. The samples were incubated for 20 min, before 0.8 mL of ethyl-*β*-8′-apo-carotenotate (Sigma Chemical Co., St. Louis, MO, USA) was added as an internal standard to establish extraction efficiency. For pigment extraction, 2.5 mL of tetrahydrofluran was added to the sample. Using a Potter-Elvehjem tissue grinding tube (Kontes, Vineland, NJ, USA), the samples was homogenized in an ice bath to dissipate heat generated from maceration. The tubes were then centrifuged in a clinical centrifuge (Centrific Model 225, Fisher Scientific, Pittsburg, PA, USA) for 3 min at 500*g*_*n*_. The supernatant was removed, and the pellet was rehydrated with 2.0 mL tetrahydrofluran. This procedure was repeated twice more until the supernatant was colorless. The combined supernatants were reduced to 0.5 mL under a stream of nitrogen gas and brought to a final volume of 5 mL with methanol. The samples were then filtered through a 0.2 *μ*m Econofilter PTFE 25/20 polytetrafluoroethylene filter (Agilent Technologies, Wilmington, DE, USA) using a 5 mL syringe. A 1.5 mL aliquot was put into an amber vial and capped prior to high performance liquid chromatography (HPLC) analysis.

**Figure 1:**
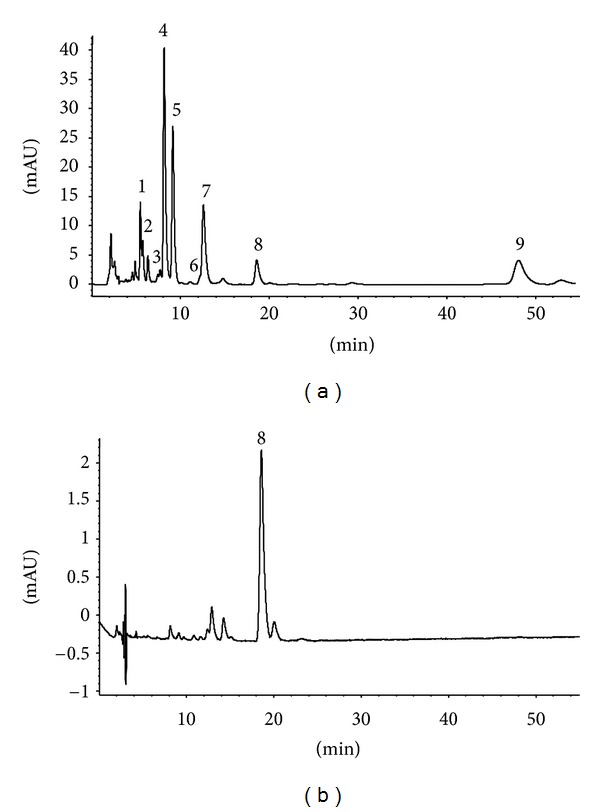
HPLC chromatogram of *Allium fistulosum *L. leaf (a) and pseudostem (b) tissues at 453 nm. Retention times (min) for the pigments were (1) violaxanthin, 5.52 min; (2) neoxanthin, 5.81 min; (3) antheraxanthin, 7.59 min; (4) chlorophyll *b*, 8.51 min; (5) lutein, 9.33 min; (6) zeaxanthin, 11.31 min; (7) chlorophyll *a*, 13.90 min; (8) ethyl-**β**-8′-apo-carotenoate (internal standard), 19.32 min; and (9) **β**-carotene, 48.46 min. HPLC conditions are described in the text.

An Agilent 1200 series HPLC unit with a photodiode array detector (Agilent Technologies, Palo Alto, CA, USA) was used for pigment separation (Figure 1). The column used was a 250 × 4.6 mm i.d., 5 **μ**m analytical scale polymeric RP-C_30_, with a 10 × 4.0 mm i.d. guard cartridge and holder (ProntoSIL, MAC-MOD Analytical Inc., Chadds Ford, PA, USA), which allowed for effective separation of chemically similar compounds. The column was maintained at 30°C using a thermostated column compartment. All separations were achieved isocratically using a binary mobile phase of 11% methyl *tert*-butyl ether (MTBE), 88.99% MeOH, and 0.01% triethylamine (TEA) (v/v/v). The flow rate was 1.0 mL/min, with a run time of 53 min. There was a 2 min equilibration prior to the next injection. Eluted compounds from a 10 *μ*L injection loop were detected at 453 nm (carotenoids, internal standard, chlorophyll *b*) and 652 nm (chlorophyll *a*). Data were collected, recorded, and integrated using ChemStation Software (Agilent Technologies). Peak assignments for each pigment were performed by comparing retention times and line spectra obtained from the photodiode array detection using external standards. Standards included antheraxanthin, neoxanthin, lutein, violaxanthin, zeaxanthin, and **β**-carotene, chlorophyll *a* and chlorophyll *b* (ChromaDex Inc., Irvine, CA, USA). The concentrations of the external standards were determined spectrophotometrically using a procedure by Davies and Köst [21]. Pigment data is presented on a fresh mass (FM) basis.

### 2.3. Statistical Analyses

Statistical analyses were completed using the GLM procedure of SAS (v. 9.1, SAS Institute, Cary, NC, USA). Cultigen means within each treatment were separated by least significant difference (LSD) at *α* = 0.05. Differences between cultigens means between treatments were detected by using Student's *t*-test (*P* = 0.05) using JMP (v 7.0.1, SAS Institute).

## 3. Results and Discussion

### 3.1. Shoot Tissue Biomass

Significant differences were found among cultigens (*F* = 6.67, *P* < 0.001) for shoot tissue height, but no differences were found between the UV-B treatments or the interaction between the cultigen and UV-B radiation treatment (Table 1). Only one cultigen (GA-C 76) differed significantly between UV-B radiation treatments for shoot tissue height. “Long White Bunching” demonstrated the greatest growth in shoot tissue height under both UV-B radiation treatments, while “G 30393-06GI” had the shortest final shoot tissue height. There were differences in shoot tissue FM between UV-B radiation treatments (*F* = 238.10, *P* < 0.001) and among cultigens (*F* = 11.09, *P* < 0.001), but no difference in the treatment by cultigen interaction (Table 1). “Deep Purple,” “Feast,” “GA-C 76,” “Ishikura Improved F1,” “Improved Beltsville Bunching,” “Jionji,” “Long White Bunching,” “Parade,” “Performer,” “Pesoenyj,” “Shounan,” “White Spear,” “274254-05GI,” and “G 30393-06GI” all showed decreases in shoot tissue FM with exposure to the UV-B radiation treatment. Significant decreases in shoot tissue biomass from the UV-B treatment would indicate a radiational stress had occurred in the bunching onion cultigens in the current study. The cultigens with the greatest shoot tissue FM accumulations were “Long White Bunching” and “Improved Beltsville Bunching” (Table 1).

### 3.2. Shoot Tissue Carotenoid Pigment Concentrations

No carotenoid pigments were measured in the pseudostem tissues of any of the bunching onion cultigens (Figure 1; data not shown). Kopsell et al. [5] also reported no carotenoid pigmentation present in bunching onion pseudostem tissues. Shoot tissue zeaxanthin differed significantly among the bunching onion cultigens (*F* = 4.07; *P* < 0.001) (Table 2). However, there were no significant changes in shoot tissue zeaxanthin in response to UV-B treatment, or the interaction of the UV-B treatments and cultigens. Only the cultigens of “G 30393-06GI” and “Feast” showed an increase in shoot tissue zeaxanthin under the supplemental UV-B radiation, as compared to control. The ranges of zeaxanthin concentrations in the bunching onions under supplemental UV-B were from 0.08 mg/100 g FM for “Deep Purple” and “White Spear” to 0.16 mg/100 g FM for “Improved Beltsville Bunching”. Cultigen “Pesoenyj” had the highest concentration of zeaxanthin among plants grown without supplemental UV radiation at 0.19 mg/100 g FM, while “Feast” and “Evergreen Hardy White” had the lowest zeaxanthin concentrations at 0.07 mg/100 g FM. Increases in zeaxanthin could be an indication that the plants experienced radiational stress from the UV-B treatment. Plant responses through increased zeaxanthin concentrations would be expected to help dissipate excess energy from the photosystems [7].

Shoot tissue violaxanthin responded significantly to both UV-B radiation treatment (*F* = 6.76; *P* = 0.0109) and cultigen (*F* = 4.42, *P* < 0.001), but not to the interaction between treatment and cultigen (Table 2). Many of the bunching onion cultigens showed higher concentrations of violaxanthin in response to UV-B radiational supplementation. However, only one cultigen had significant increases in violaxanthin concentrations (GA-C 76) in response to UV-B radiation treatment. Increases in violaxanthin in bunching onions grown under UV-B radiation may suggest that these cultigens may not be as susceptible to UV-B radiational damage as the other cultigens. Violaxanthin concentrations under supplemental UV-B radiation ranged from 2.04 mg/100 g FM for “GA-C 76” to 0.59 mg/100 g FM for “Performer.” Cultigen “Pesoenyj” had the highest concentrations of violaxanthin (2.35 mg/100 g FM) for bunching onions grown without supplemental UV-B radiation, while “G 30393-06GI” had the lowest violaxanthin concentrations (0.53 mg/100 g FM).

Antheraxanthin, the intermediate compound in xanthophyll cycle, responded significantly to changes in UV-B radiation treatments (*F* = 16.61; *P* < 0.0001) and cultigens (*F* = 4.68; *P* < 0.001). The majority of cultigens had higher antheraxanthin concentrations in response to the UV-B radiation treatment; however, no cultigens had significantly higher levels as compared to the control treatment (Table 2). The ranges for antheraxanthin concentrations in bunching onions grown under UV-B radiation treatment were from 1.38 mg/100 g FM for “Pesoenyj” to 0.79 mg/100 g FM for “274254-05GI.” In the plants grown without UV-B radiation, “Pesoenyj” had the highest antheraxanthin concentrations (1.35 mg/100 g FM), while “Ishikura Improved F1” had the lowest concentrations (0.59 mg/100 g FM). While changes in this compound cannot directly tell which way the xanthophyll cycle is fluxing, increases or decreases may help predict potential energy flow.

Neoxanthin concentrations responded significantly to UV-B radiation treatment (*F* = 12.13; *P* = 0.0008), cultigen (*F* = 3.20; *P* = 0.0003), and the interaction of UV radiation treatment and cultigen (*F* = 2.27; *P* = 0.0092). There were significant increases in neoxanthin from the UV-B treatment for the cultigens “Feast,” “GA-C 76,” and “G 30393-06GI” when compared to the control treatment (Table 3). “Feast” showed the highest concentrations of neoxanthin under UV-B radiation treatment (1.86 mg/100 g FM), while “Deep Purple” had the lowest concentration of neoxanthin (0.73 mg/100 g FM). “Pesoenyj” showed the highest neoxanthin concentration (1.96 mg/100 g FM) compared to the other cultigens grown under the control treatment. “Hardy Evergreen White” had the lowest of all of the cultigens not grown under supplemental UV-B radiation at 0.40 mg/100 g FM.

The bunching onions showed significant changes in lutein in response to UV-B treatment (*F* = 17.89; *P* < 0.0001) and cultigen (*F* = 2.34; *P* = 0.0070). The majority of cultigens had higher lutein concentrations in response to the UV-B radiation treatment; however, only “Feast” and “GA-C 76” had significantly higher lutein (Table 3). “Pesoenyj” had the highest concentrations of lutein both with and without supplemental UV-B radiation at 8.01 and 9.23 mg/100 g FM, respectively. “Deep Purple” had the lowest concentration of lutein among bunching onions grown with supplemental UV-B radiation at 5.04 mg/100 g FM, and “Feast” had the lowest amount of lutein for bunching onions grown without supplemental UV-B radiation at 4.11 mg/100 g FM. Lutein acts as an accessory pigment and is the predominant carotenoid in photosystem (PS) II [7]. Research shows UV radiation will impact PSII functioning to a greater extent than PSI [22]. Therefore, increases in lutein concentrations for the cultigens in the current study may indicate increased radiational stress within PSII from the supplemental UV-B treatment.

Concentrations of *β*-carotene showed no changes in response to UV treatment or cultigen (Table 3). “Pesoenyj” had the highest concentrations of *β*-carotene in bunching onions grown without UV-B radiation, and “Ishikura Improved F1” had the lowest concentrations. The range of shoot tissue *β*-carotene levels for cultigens grown under supplemental UV-B radiation were 2.80 mg/100 g FM for “Shounan” to 0.88 mg/100 g FM for “Evergreen Hardy White.” For the cultigens that were not grown under supplemental UV-B radiation, the ranges for *β*-carotene concentration were 3.45 mg/100 g FM (Pesoenyj) and 0.64 mg/100 g FM (Evergreen Hardy White). Reported mean value for *β*-carotene in shoot tissues of *A. fistulosum* is 0.60 mg/100 g FM, while the mean values for lutein and zeaxanthin are 1.14 mg/100 g FM [23]. Umehara et al. [24] reported *β*-carotene values in the leaves of* A. fistulosum* L. cultigen “Kujyoasagikei” to be 4.63 mg/100 g FM. *β*-carotene is an accessory pigment and is the predominant carotenoid in PSI. *β*-carotene is present in PSII, but mostly in regions around the reaction center [7]. Since there were no impacts on *β*-carotene concentrations in the current study, it is possible that PSI is not under as much stress from the UV-B treatments imposed in this study [22].

The xanthophyll cycle pigments (zeaxanthin, antheraxanthin, and violaxanthin) are important for the dissipation of excess absorbed light, performed almost exclusively by ZEA. Photosynthetic rates are reduced under many environmental stressors, which increase the need for dissipation of excess absorbed light energy [7]. The ratio of zeaxanthin + antheraxanthin to zeaxanthin + antheraxanthin + violaxanthin (ZA/ZAV) responded significantly to cultigen (*F* = 3.01; *P* = 0.0006), but not to UV-B radiation treatment or the interaction between treatment and cultigen. Significant increases in response to supplemental UV-B were found for “Pesoenyj.” “G 30393-06GI” had the highest ZA/ZAV ratio of cultigens grown under supplemental UV-B radiation, and “Ishikura Improved F1” had the lowest ZA/ZAV ratio at 0.34. For the cultigens not grown under UV-B radiation, “Feast” had the highest ZA/ZAV ratio at 0.65, while “Jionji Negi” had the lowest ZA/ZAV ratio at 0.35 (Table 4). Changes in the ZA/ZAV ratio can identify fluxes within the xanthophyll energy dissipation cycle. An increase in ZA/ZAV ratio shows a decrease in violaxanthin, which could mean these compounds are undergoing deepoxydation because of high light energy [7]. A study by Niyogi et al. [25] helped demonstrate the importance of this photoprotective mechanistic cycle. In this study, mutant *Arabidopsis thaliana* was unable to undergo deepoxydation and converts violaxanthin to zeaxanthin, which resulted in an increased sensitivity to higher light levels. While the Niyogi et al. [25] study did not specifically look at how UV-B radiation affected xanthophyll cycle functioning, energy from UV wavelengths is higher than energy from PAR wavelengths and could be expected to change the flux between the xanthophyll pigments.

Kopsell et al. [5] grew many of the same bunching onion cultigens under field conditions in Knoxville, TN, USA, and Geneva, NY, USA, and reported similar levels of shoot tissue *β*-carotene and neoxanthin as found in the current study; however, values for violaxanthin, antheraxanthin, lutein, chlorophyll *a*, and chlorophyll *b* were much higher in the current study than previously reported. Differences in shoot tissue pigments for cultigens among the two studies may be attributed to differences in growing conditions (field versus glasshouse) and the time of year the cultigens were evaluated (summer versus winter).

Epidemiological data supports the positive association between increased dietary intake of plant foods high in carotenoids and greater carotenoid tissue concentrations with lower risks of certain chronic diseases. Many of these disease suppressing abilities can be attributed to the antioxidant properties of carotenoids. One of the most important physiological functions of carotenoids in human nutrition is as vitamin A precursors. Provitamin A carotenoid compounds (*β*-carotene, *α*-carotene, and cryptoxanthins) support the maintenance of healthy epithelial cell differentiation, normal reproductive performance, and visual functions [26]. Both provitamin A carotenoids and nonprovitamin A carotenoids (lutein, zeaxanthin, and lycopene) function as free radical scavengers, enhance the immune response, suppress cancer development, and protect eye tissues [27]. Humans cannot synthesize carotenoids and therefore must rely on dietary sources to provide sufficient levels. Studies indicate that high intakes of a variety of vegetables, providing a mixture of carotenoids, were more strongly associated with reduced cancer and eye disease risk than intake of individual carotenoid supplements [28]. There is clear evidence that cultural practices that maintain or enhance tissue carotenoid levels would be beneficial to humans when regularly consumed in the diet.

### 3.3. Shoot Tissue Chlorophyll Pigment Concentrations

No chlorophyll pigments were measured in the pseudostem tissues of any of the bunching onion cultigens (Figure 1; data not shown). Chlorophyll *a* responded significantly to UV radiation treatments (*F* = 4.35; *P* = 0.0398), but not to cultigens or the interaction between treatment and cultigen. “Feast” had the highest concentration of chlorophyll *a* at 59.56 mg/100 g FM for cultigens grown under supplemental UV-B radiation, while “Deep Purple” had the lowest at 27.75 mg/100 g FM. For the cultigens grown without supplemental UV-B radiation, “Pesoenyj” had the highest concentration of chlorophyll *a* at 63.27 mg/100 g FM, while “Evergreen Hardy White” had the lowest at 16.52 mg/100 g FM (Table 5). Values for chlorophyll *a* for cultigens are in close agreement with Dissanayake et al. [29] who reported values of ~75.00 mg chlorophyll *a*/100 g FM for the *A. fistulosum *cultigen “Kujyo-hoso.”

The bunching onions showed significant differences in chlorophyll *b* caused by UV-B treatment (*F* = 19.04; *P* < 0.0001) and cultigen (*F* = 2.08; *P* = 0.0179), but there were no influences from their interaction. Values for chlorophyll *b* for cultigens are in close agreement with Dissanayake et al. [29] who reported values of ~17.00 mg chlorophyll *b*/100 g FM for the *A. fistulosum *cultigen “Kujyo-hoso.” Significant increases in chlorophyll *b* in response to UV-B radiation were found for cultigens “Feast,” “GA-C 76,” and “Shounan” (Table 5). The concentrations of chlorophyll *b* for cultigens grown under supplemental UV-B radiation ranged from 29.24 mg/100 g FM for “GA-C 76” to 18.49 mg/100 g FM for “Improved Beltsville Bunching.” For cultigens grown without supplemental UV-B radiation, chlorophyll *b* concentrations ranged from 29.74 mg/100 g FM for “Pesoenyj” to 15.78 mg/100 g FM for “Improved Beltsville Bunching.”

Concentrations of total chlorophyll (chlorophyll *a* + *b*) in bunching onions were found to differ between UV-B treatments (*F* = 6.82; *P* = 0.0105), but not among cultigens. “Feast” and “GA-C 76” were the only bunching onion cultigens to show differences between UV-B treatments (Table 5). Total chlorophyll concentrations ranged from 88.82 mg/100 g FM for “Feast” to 45.62 mg/100 g FM for “Zhang Qui Da Cong” for bunching onions grown under supplemental UV-B radiation. For the plants grown without UV-B radiation, ranges from total chlorophyll varied from 93.01 mg/100 g FM for “Pesoenyj” to 34.74 mg/100 g FM for “Evergreen Hardy White.”

The ratio of chlorophyll *a* to chlorophyll *b* in the bunching onions showed significant changes based on cultigen (*F* = 2.26; *P* = 0.0094), but not for UV-B radiation treatments. In general, cultigens were evenly divided in their responses to UV-B radiation, with half the cultigens displaying higher chlorophyll *a*/chlorophyll *b* under the supplemental UV-B radiation treatment (Table 4). However, only the cultigen “Feast” had a significantly higher chlorophyll *a*/chlorophyll *b* ratio under UV-B radiation. “Long White Bunching” had the highest chlorophyll *a*/chlorophyll *b* ratio in the bunching onions grown without supplemental UV-B at 2.25, and “GA-C 76” had the lowest ratio at 0.91. Under UV-B radiation treatment, “Feast” has the highest chlorophyll *a*/chlorophyll *b* ratio at 2.14, while “Improved Beltsville Bunching” has the lowest ratio at 1.04.

### 3.4. Shoot Tissue Photochemical Efficiency (*F*_*v*_/*F*_*m*_)

Photochemical efficiency (*F*_*v*_/*F*_*m*_) showed significant differences between UV treatments (*F* = 13.89, *P* = 0.0003) and cultigen (*F* = 2.11, *P* = 0.0152), but no difference due to treatment and cultigen interaction (data not shown). Values for *F*_*v*_/*F*_*m*_ for all of the cultigens evaluated in the study averaged 0.82. One previous study by Tsormpatsidis et al. [30] showed that while “Lollo Rosso” lettuce (*Lactuca sativa* L.) had decreased vegetative growth under UV light treatments, there was no difference in photochemical efficiency. By contrast, when the agronomic crop wheat was exposed to UV radiation, decreases in *F*_*v*_/*F*_*m*_ occurred under the UV light treatment [31]. None of the cultigens in this study showed differences in *F*_*v*_/*F*_*m*_; however, most of the cultigens differed in shoot tissue fresh biomass when exposed to UV-B radiation.

## 4. Conclusion

Data from multiple studies demonstrates cultigens within a given plant species can react differently under variable stress conditions. Most often, harsh stress conditions negatively impact plant biomass. In the current study, decreases in bunching onion shoot tissue biomass confirmed that a radiational stress from the UV-B treatment had occurred. The bunching onion cultigens demonstrated genetic variability in response to UV-B radiation (Tables 1–5). Changes in plant pigments associated with light harvesting and photoprotection can be expected when bunching onion cultigens experience greater levels of UV-B radiation in the growing environment. In the current study, the cultigens with the greatest stimulation in carotenoid pigments from UV-B exposure were “Feast” and the accession G 30393-06GI. Data presented here may be valuable to improve abiotic stress tolerance to increasing UV-B radiation for specialty crop breeding programs.

## Figures and Tables

**Table 1:** Mean values^a^ for shoot tissue height (cm) and fresh biomass (g) for *Allium fistulosum* L. cultigens grown under supplemental UV-B (313 nm) light [7.0 *μ*mol·m^−2^·s^−2^ (2.68 W·m^−2^); UV-B] or UV-filtered (control) light in a glasshouse in Knoxville, TN, USA (35°96′N Lat.).

Cultigen	Shoot tissue height (cm)			Shoot tissue fresh biomass (g)		
	UV-B	Control	Pr > |*t*|^b^	UV-B	Control	Pr > |*t*|
Deep Purple	44.71 ± 5.49	45.19 ± 4.52	ns	64.54 ± 17.02	97.80 ± 17.44	*P* = 0.034
Evergreen Hardy White	39.11 ± 1.96	41.05 ± 5.38	ns	38.65 ± 7.80	70.78 ± 8.08	ns
Feast	38.58 ± 3.24	39.96 ± 2.40	ns	48.79 ± 15.99	90.46 ± 13.85	*P* = 0.008
GA-C 76	38.82 ± 1.91	44.13 ± 3.70	*P* = 0.043	41.90 ± 2.66	73.00 ± 15.53	*P* = 0.008
Ishikura improved F1	39.63 ± 1.12	41.17 ± 3.13	ns	54.93 ± 9.22	99.80 ± 9.47	*P* = 0.001
Improved Beltsville Bunching	44.26 ± 1.69	48.06 ± 2.19	ns	76.86 ± 9.82	110.42 ± 19.39	*P* = 0.021
Jionji Negi	36.20 ± 1.82	38.63 ± 1.54	ns	38.05 ± 4.73	62.04 ± 8.23	*P* = 0.002
Long White Bunching	49.68 ± 2.09	50.38 ± 4.62	ns	74.07 ± 8.73	116.01 ± 20.24	*P* = 0.009
Parade	42.13 ± 2.60	36.03 ± 9.40	ns	53.89 ± 10.73	90.81 ± 16.87	*P* = 0.010
Performer	39.37 ± 2.36	39.42 ± 3.10	ns	54.74 ± 11.08	86.25 ± 16.66	*P* = 0.020
Pesoenyj	39.44 ± 4.23	44.54 ± 4.03	ns	29.22 ± 9.30	58.76 ± 6.37	*P* = 0.002
Shounan	36.72 ± 3.02	37.14 ± 2.54	ns	39.17 ± 3.56	63.32 ± 4.02	*P* = 0.001
White Spear	40.06 ± 2.79	42.42 ± 1.72	ns	53.16 ± 14.41	87.42 ± 10.58	*P* = 0.009
Zhang Qui Da Cong	36.62 ± 2.88	37.87 ± 3.80	ns	53.57 ± 19.51	84.72 ± 16.67	ns
274254-05GI	42.48 ± 1.86	43.79 ± 4.40	ns	47.63 ± 4.34	84.65 ± 6.98	*P* = 0.001
G 30393-06GI	36.62 ± 3.40	35.67 ± 1.31	ns	50.96 ± 8.63	81.69 ± 12.01	*P* = 0.006
LSD_0.05_^c^	5.77	5.80		10.72	19.33	

^a^Composition of *n* = 6 plant samples from 4 replications ± standard deviation. ^b^Significance based on paired Student's* t*-test among treatments; ns: not significant. ^c^LSD for differences between cultivar means *α* = 0.05.

**Table 2:** Mean values^a^ for shoot tissue zeaxanthin, violaxanthin, and antheraxanthin (mg/100 g fresh mass) for *Allium fistulosum* L. cultigens grown under supplemental UV-B (313 nm) light [7.0 μmol·m^−2^·s^−2^ (2.68 W·m^−2^); UV-B] or UV-filtered (control) light in a glasshouse in Knoxville, TN, USA (35°96′N Lat.).

Cultigen	Zeaxanthin			Violaxanthin			Antheraxanthin		
	UV-B	Control	Pr > |*t*|^b^	UV-B	Control	Pr > |*t*|	UV-B	Control	Pr > |*t*|
mg/100 g fresh mass									

Deep Purple	0.08 ± 0.02	0.10 ± 0.03	ns	1.25 ± 0.36	0.88 ± 0.42	ns	1.07 ± 0.31	0.78 ± 0.23	ns
Evergreen Hardy White	0.10 ± 0.03	0.07 ± 0.02	ns	1.73 ± 0.61	1.20 ± 0.25	ns	1.03 ± 0.18	0.78 ± 0.14	ns
Feast	0.11 ± 0.01	0.07 ± 0.01	*P* = 0.010	1.35 ± 0.82	0.66 ± 0.58	ns	1.15 ± 0.27	0.92 ± 0.19	ns
GA-C 76	0.12 ± 0.02	0.08 ± 0.02	ns	2.04 ± 0.19	1.34 ± 0.20	*P* = 0.002	1.92 ± 0.65	1.27 ± 0.47	ns
Ishikura Improved F1	0.10 ± 0.05	0.09 ± 0.02	ns	1.75 ± 0.94	1.10 ± 0.38	ns	0.81 ± 0.50	0.59 ± 0.13	ns
Improved Beltsville Bunching	0.16 ± 0.02	0.15 ± 0.04	ns	1.62 ± 0.54	1.25 ± 0.14	ns	0.99 ± 0.38	0.72 ± 0.21	ns
Jionji Negi	0.12 ± 0.06	0.13 ± 0.03	ns	1.54 ± 0.25	1.51 ± 0.35	ns	1.20 ± 0.25	0.78 ± 0.50	ns
Long White Bunching	0.12 ± 0.03	0.11 ± 0.03	ns	0.89 ± 0.18	0.81 ± 0.42	ns	0.82 ± 0.13	0.89 ± 0.14	ns
Parade	0.10 ± 0.02	0.11 ± 0.02	ns	1.23 ± 0.50	1.02 ± 0.61	ns	0.86 ± 0.16	0.79 ± 0.24	ns
Performer	0.09 ± 0.02	0.10 ± 0.02	ns	0.59 ± 0.52	1.45 ± 0.60	ns	0.87 ± 0.06	0.85 ± 0.32	ns
Pesoenyj	0.12 ± 0.03	0.19 ± 0.06	ns	1.93 ± 0.33	2.35 ± 0.82	ns	1.38 ± 0.30	1.35 ± 0.52	ns
Shounan	0.13 ± 0.05	0.09 ± 0.01	ns	1.87 ± 0.81	1.04 ± 0.36	ns	1.18 ± 0.40	0.63 ± 0.30	ns
White Spear	0.08 ± 0.02	0.08 ± 0.03	ns	1.00 ± 0.60	0.77 ± 0.47	ns	0.74 ± 0.13	0.60 ± 0.09	ns
Zhang Qui Da Cong	0.11 ± 0.04	0.12 ± 0.02	ns	1.29 ± 0.48	1.30 ± 0.41	ns	0.81 ± 0.30	0.74 ± 0.06	ns
274254-05GI	0.13 ± 0.04	0.15 ± 0.03	ns	1.64 ± 0.42	1.27 ± 0.52	ns	0.79 ± 0.14	0.71 ± 0.33	ns
G 30393-06GI	0.13 ± 0.03	0.08 ± 0.02	*P* = 0.016	0.60 ± 0.51	0.50 ± 0.43	ns	1.07 ± 0.35	0.67 ± 0.14	ns
LSD_0.05_^c^	ns	0.04		0.74	0.70		0.44	0.44	

^a^Composition of *n* = 6 plant samples from 4 replications ± standard deviation. ^b^Significance based on paired Student's* t*-test among treatments; ns: not significant. ^c^LSD for differences between cultivar means *α* = 0.05.

**Table 3:** Mean values^a^ for shoot tissue neoxanthin, lutein, and *β*-carotene (mg/100 g fresh mass) for *Allium fistulosum* L. cultigens grown under supplemental UV-B (313 nm) light [7.0 μmol·m^−2^·s^−2^ (2.68 W·m^−2^); UV-B] or UV-filtered (control) light in a glasshouse in Knoxville, TN, USA (35°96′N Lat.).

Cultigen	Neoxanthin			Lutein			*β*-carotene		
	UV-B	Control	Pr > |*t*|^b^	UV-B	Control	Pr > |*t*|	UV-B	Control	Pr > |*t*|
mg/100 g fresh mass									

Deep Purple	0.73 ± 0.51	1.04 ± 0.59	ns	5.04 ± 1.48	5.38 ± 0.73	ns	1.07 ± 0.81	1.09 ± 0.30	ns
Evergreen Hardy White	0.74 ± 0.47	0.40 ± 0.18	ns	7.10 ± 2.86	5.10 ± 1.49	ns	0.88 ± 0.71	0.64 ± 0.23	ns
Feast	2.09 ± 0.48	0.79 ± 0.57	*P* = 0.013	7.66 ± 0.90	4.11 ± 0.54	*P* = 0.001	1.85 ± 0.85	1.04 ± 0.74	ns
GA-C 76	1.53 ± 0.32	0.66 ± 0.18	*P* = 0.003	7.66 ± 0.38	5.57 ± 0.67	*P* = 0.002	1.48 ± 0.27	1.17 ± 0.09	ns
Ishikura Improved F1	0.63 ± 0.43	0.63 ± 0.30	ns	6.35 ± 3.18	4.80 ± 1.20	ns	2.20 ± 2.59	0.78 ± 0.41	ns
Improved Beltsville Bunching	0.82 ± 0.89	0.60 ± 0.16	ns	6.95 ± 1.34	5.62 ± 0.65	ns	1.64 ± 0.95	1.39 ± 0.30	ns
Jionji Negi	0.92 ± 0.25	0.91 ± 0.36	ns	7.35 ± 1.65	6.21 ± 1.49	ns	1.74 ± 1.20	2.29 ± 1.32	ns
Long White Bunching	1.76 ± 0.26	1.47 ± 0.35	ns	6.00 ± 0.58	5.05 ± 1.17	ns	1.69 ± 0.29	1.94 ± 0.56	ns
Parade	1.46 ± 0.81	0.87 ± 0.36	ns	6.36 ± 1.17	6.04 ± 1.47	ns	1.26 ± 0.14	2.54 ± 1.23	ns
Performer	1.14 ± 0.90	0.75 ± 0.47	ns	6.33 ± 0.96	5.60 ± 2.36	ns	1.28 ± 0.48	2.38 ± 1.65	ns
Pesoenyj	1.03 ± 0.19	1.96 ± 0.78	ns	8.01 ± 1.21	9.23 ± 2.59	ns	1.87 ± 0.20	3.45 ± 2.39	ns
Shounan	1.32 ± 0.71	0.54 ± 0.32	ns	7.66 ± 2.83	5.03 ± 1.26	ns	2.80 ± 1.46	1.10 ± 0.54	ns
White Spear	1.21 ± 0.62	0.93 ± 0.63	ns	6.08 ± 0.94	4.70 ± 1.37	ns	1.49 ± 0.51	1.08 ± 0.60	ns
Zhang Qui Da Cong	0.75 ± 0.42	0.65 ± 0.29	ns	5.65 ± 1.44	5.31 ± 1.47	ns	1.05 ± 0.24	1.20 ± 0.57	ns
274254-05GI	0.84 ± 0.37	0.72 ± 0.45	ns	6.18 ± 0.79	5.33 ± 1.82	ns	1.81 ± 0.86	1.86 ± 1.57	ns
G 30393-06GI	1.86 ± 0.44	0.85 ± 0.62	*P* = 0.040	6.02 ± 1.18	4.42 ± 0.69	ns	1.86 ± 0.77	1.28 ± 0.67	ns
LSD_0.05_^c^	0.78	0.67		ns	2.14		ns	1.54	

^a^Composition of *n* = 6 plant samples from 4 replications ± standard deviation. ^b^Significance based on paired Student's* t*-test among treatments; ns: not significant. ^c^LSD for differences between cultivar means *α* = 0.05.

**Table 4:** Mean values^a^ for the ratio of zeaxanthin + antheraxanthin to zeaxanthin + antheraxanthin + violaxanthin (Z + A/A + Z + V) and the ratio of chlorophyll *a* to chlorophyll *b* (chlorophyll *a*/chlorophyll *b*) in shoot tissues for *Allium fistulosum* L. cultigens grown under supplemental UV-B (313 nm) light [7.0 μmol·m^−2^·s^−2^ (2.68 W·m^−2^); UV-B] or UV-filtered (control) light in a glasshouse in Knoxville, TN, USA (35°96′N Lat.).

Cultigen	Z + A/A + Z + V			Chlorophyll *a*/chlorophyll *b *		
	UV-B	Control	Pr > |*t*|^b^	UV-B	Control	Pr > |*t*|
Deep Purple	0.48 ± 0.01	0.51 ± 0.19	ns	1.25 ± 0.90	1.50 ± 0.23	ns
Evergreen Hardy White	0.41 ± 0.06	0.41 ± 0.01	ns	1.15 ± 0.56	0.91 ± 0.16	ns
Feast	0.51 ± 0.16	0.65 ± 0.21	ns	2.14 ± 0.43	1.07 ± 0.63	*P* = 0.031
GA-C 76	0.49 ± 0.07	0.49 ± 0.10	ns	1.58 ± 0.18	0.93 ± 0.51	ns
Ishikura Improved F1	0.34 ± 0.03	0.40 ± 0.08	ns	1.69 ± 1.06	1.45 ± 0.70	ns
Improved Beltsville Bunching	0.41 ± 0.03	0.41 ± 0.07	ns	1.04 ± 0.92	1.29 ± 0.50	ns
Jionji Negi	0.46 ± 0.07	0.35 ± 0.15	ns	1.85 ± 0.70	2.21 ± 0.50	ns
Long White Bunching	0.52 ± 0.04	0.58 ± 0.17	ns	1.86 ± 0.06	2.25 ± 0.68	ns
Parade	0.45 ± 0.10	0.50 ± 0.24	ns	1.77 ± 0.33	2.16 ± 0.48	ns
Performer	0.66 ± 0.17	0.40 ± 0.02	ns	1.60 ± 0.11	2.07 ± 1.19	ns
Pesoenyj	0.44 ± 0.03	0.39 ± 0.01	*P* = 0.022	1.40 ± 0.42	1.95 ± 0.80	ns
Shounan	0.42 ± 0.03	0.40 ± 0.03	ns	1.78 ± 0.52	1.08 ± 0.53	ns
White Spear	0.50 ± 0.21	0.51 ± 0.22	ns	1.46 ± 0.42	1.08 ± 0.62	ns
Zhang Qui Da Cong	0.42 ± 0.05	0.41 ± 0.08	ns	1.11 ± 0.50	1.32 ± 0.46	ns
274254-05GI	0.36 ± 0.06	0.40 ± 0.00	ns	1.80 ± 0.71	2.14 ± 0.81	ns
G 30393-06GI	0.69 ± 0.20	0.55 ± 0.12	ns	1.84 ± 0.31	1.32 ± 0.64	ns
LSD_0.05_^c^	0.15	0.20		ns	0.96	

^a^Composition of *n* = 6 plant samples from 4 replications ± standard deviation. ^b^Significance based on paired Student's *t*-test among treatments; ns: not significant. ^c^LSD for differences between cultivar means *α* = 0.05.

**Table 5:** Mean values^a^ for shoot tissue chlorophyll *a*, chlorophyll *b*, and total chlorophyll (chlorophyll *a* + chlorophyll *b*) (mg/100 g fresh mass) for *Allium fistulosum* L. cultigens grown under supplemental UV-B (313 nm) light [7.0 *μ*mol·m^−2^·s^−2^ (2.68 W·m^−2^); UV-B] or UV-filtered (control) light in a glasshouse in Knoxville, TN, USA (35°96′N Lat.).

Cultigen	Chlorophyll *a *			Chlorophyll *b *			Total chlorophyll		
	UV-B	Control	Pr > |*t*|^b^	UV-B	Control	Pr > |*t*|	UV-B	Control	Pr > |*t*|
mg/100 g fresh mass									

Deep Purple	27.8 ± 22.1	25.3 ± 5.6	ns	20.6 ± 4.4	16.9 ± 2.4	ns	48.3 ± 25.6	42.1 ± 7.5	ns
Evergreen Hardy White	31.4 ± 21.1	16.5 ± 3.2	ns	25.5 ± 5.1	18.2 ± 0.7	ns	56.9 ± 26.1	34.7 ± 3.5	ns
Feast	59.6 ± 20.3	19.0 ± 10.5	*P* = 0.012	27.3 ± 4.0	17.7 ± 1.9	*P* = 0.005	86.8 ± 24.3	36.7 ± 11.0	*P* = 0.009
GA-C 76	47.0 ± 16.4	17.2 ± 10.6	ns	29.2 ± 7.1	18.1 ± 1.3	*P* = 0.021	76.2 ± 23.3	35.4 ± 11.5	*P* = 0.020
Ishikura Improved F1	49.9 ± 53.6	23.1 ± 5.9	ns	25.6 ± 11.6	17.6 ± 4.7	ns	74.5 ± 65.1	40.7 ± 2.6	ns
Improved Beltsville Bunching	23.9 ± 28.4	20.6 ± 8.9	ns	18.5 ± 7.6	15.8 ± 1.0	ns	42.4 ± 36.0	36.1 ± 9.8	ns
Jionji Negi	48.3 ± 23.4	47.2 ± 18.9	ns	25.5 ± 3.1	20.9 ± 4.8	ns	73.8 ± 26.0	68.0 ± 23.4	ns
Long White Bunching	36.9 ± 6.6	37.2 ± 7.4	ns	19.7 ± 2.8	17.1 ± 2.7	ns	56.5 ± 9.2	54.3 ± 6.3	ns
Parade	38.6 ± 13.2	44.4 ± 18.8	ns	21.2 ± 4.1	19.9 ± 4.4	ns	59.8 ± 17.2	64.3 ± 23.0	ns
Performer	36.8 ± 3.1	49.6 ± 31.9	ns	23.0 ± 2.1	20.9 ± 7.1	ns	59.8 ± 4.8	70.5 ± 38.9	ns
Pesoenyj	36.8 ± 10.2	63.3 ± 36.9	ns	26.3 ± 2.4	29.7 ± 8.7	ns	26.3 ± 2.4	93.0 ± 45.6	ns
Shounan	44.3 ± 24.9	20.6 ± 19.1	ns	24.3 ± 8.3	16.6 ± 7.6	ns	68.6 ± 32.6	37.2 ± 26.7	ns
White Spear	30.4 ± 9.9	18.2 ± 9.6	ns	20.8 ± 1.9	17.8 ± 1.0	*P* = 0.033	51.2 ± 10.7	35.4 ± 8.9	ns
Zhang Qui Da Cong	25.3 ± 18.3	27.3 ± 13.5	ns	20.4 ± 7.4	19.3 ± 4.2	ns	45.6 ± 25.6	47.1 ± 17.6	ns
274254-05GI	36.6 ± 23.1	40.0 ± 18.1	ns	18.9 ± 5.3	18.7 ± 4.9	ns	55.5 ± 28.3	58.6 ± 21.5	ns
G 30393-06GI	39.8 ± 11.3	22.6 ± 13.8	ns	21.3 ± 4.1	17.0 ± 3.7	ns	61.1 ± 15.1	39.6 ± 16.0	ns
LSD_0.05_^c^	ns	26.0		ns	6.8		ns	31.6	

^a^Composition of *n* = 6 plant samples from 4 replications ± standard deviation. ^b^Significance based on paired Student's *t*-test among treatments; ns: not significant. ^c^LSD for differences between cultivar means *α* = 0.05.
